# High Catalytic Activity of Lipase from *Yarrowia lipolytica* Immobilized by Microencapsulation

**DOI:** 10.3390/ijms19113393

**Published:** 2018-10-30

**Authors:** Adejanildo da S. Pereira, Jully L. Fraga, Marianne M. Diniz, Gizele C. Fontes-Sant’Ana, Priscilla F. F. Amaral

**Affiliations:** 1 Escola de Química, Universidade Federal do Rio de Janeiro, 21941-909 Rio de Janeiro, Brazil; adejanildosp@gmail.com (A.d.S.P.); jully.lfraga@gmail.com (J.L.F.); mariannemdiniz@gmail.com (M.M.D.); 2 Instituto de Química, Departamento de Tecnologia de Processos Bioquímicos, Universidade do Estado do Rio de Janeiro, 20550-013 Rio de Janeiro, Brazil; gizele.santana@uerj.br

**Keywords:** immobilization, ionotropic gelation, sodium alginate, chitosan, calcium chloride, lipase, *Yarrowia lipolytica*

## Abstract

Microencapsulation of lipase from *Yarrowia lipolytica* IMUFRJ 50682 was performed by ionotropic gelation with sodium alginate. Sodium alginate, calcium chloride and chitosan concentrations as well as complexation time were evaluated through experimental designs to increase immobilization yield (IY) and immobilized lipase activity (ImLipA) using *p*-nitrophenyl laurate as substrate. To adjust both parameters (IY and ImLipA), the desirability function showed that microcapsule formation with 3.1%(*w*/*v*) sodium alginate, 0.19%(*w*/*v*) chitosan, 0.14 M calcium chloride, and 1 min complexation time are ideal for maximal immobilization yield and immobilized lipase activity. A nearly twofold enhancement in Immobilization yield and an increase up to 280 U/g of the lipase activity of the microcapsules were achieved using the experimental design optimization tool. Chitosan was vital for the high activity of this new biocatalyst, which could be reused a second time with about 50% of initial activity and for four more times with about 20% of activity.

## 1. Introduction

Immobilization is a powerful tool to improve enzyme properties. Enzymatic immobilization arose primarily as a response to the need of reusing expensive enzymes in industrial processes [1]. However, currently this technology not only solves the problem associated with enzyme recovery, but also, if used properly, improves many other characteristics of enzymes, such as stability, selectivity, activity, resistance to inhibitors and purity, among others [2,3].

Lipases (triacylglycerol acylhydrolases EC 3.1.1.3) are known as one of the main biocatalysts with the greatest potential in lipid technology. Lipases catalyze the hydrolysis of triglycerides into diglycerides, monoglycerides, glycerol, and free fatty acids [4]. At low water content, they also catalyze synthesis reactions generating a wide range of esters [5]. The production of lipases is carried out by means of fermentation processes, with bacteria, filamentous fungi, or yeasts. Among yeasts, *Yarrowia lipolytica* is the specie that stands out because it produces great amount of this enzyme and it has GRAS (generally recognized as safe) status, providing acceptance for use in food production [6,7]. *Y. lipolytica* is known to possess 16 paralogs of genes coding for lipase, out of which Lip2 is the main extracellular lipase secreted by this yeast [8]. The structural feature of Lip2 is characterized by the presence of a mobile subdomain, called lid, whose conformational changes control the access of substrate molecules to the catalytic center [9].

Lipases can be used in their free or immobilized forms. However, in industry, the immobilized form is preferable because it allows higher stability and, depending on the immobilization method, it can even increase lipase activity [10]. Manoel et al. [11] confirmed that lipases immobilized on octyl agarose presented their open form stabilized while the covalent preparation maintains a closing/opening equilibrium that may be modulated by altering the medium. According to the modes of interaction between enzymes and support carriers, enzyme immobilization methods can be classified into chemical or physical methods. Among the physical methods (adsorption or entrapment), entrapment can be performed by gel/fiber entrapping, by metal organic frameworks embedding and by microencapsulation [12].

Microencapsulation consists of the entrapment of the enzyme within the network of a matrix or polymer membrane. In this method, the enzymes are retained in the nets while substrates and products pass through, which avoids the contamination of the leached enzyme in the substrate solution as observed when physical adsorption is used to immobilize enzymes. Additionally, it improves stability and allows the generation of enzymatic reactions [12]. Alginate, an anionic linear copolymer composed of 1,4′-linked β-d-mannuronic acid and α-l-guluronic acid, is the most frequently used polymer for microencapsulation due to its mild gelling properties and non-toxicity [13]. Another natural polymer that has been used for microencapsulation is chitosan, which is obtained by N-deacetylation of chitin and is the second most abundant naturally occurring polymer found in the exoskeleton of marine crustaceans [14]. Chitosan has been used for several biomedical applications such as: drug, gene and vaccine delivery, tissue engineering and as biological iron chelator [14]. It acts as a polycation in solution, is readily soluble in dilute acid solutions and was used before in core-shell microcapsule technology for enzyme immobilization [15].

Even though several enzymes as well as lipases have been immobilized in alginate beads [13,16,17], no results of optimization of microencapsulation conditions of *Y. lipolytica* lipase have been found in literature. The optimization of microencapsulation conditions is essential to obtain high enzyme activity. Chitosan was tested during the formation of the microcapsules, a different approach from the coating method already used [13]. In this context, statistical experimental design was used to obtain the best conditions (alginate, chitosan and calcium chloride concentrations and complexation time) to microencapsulate the lipase produced from residual oil with *Yarrowia lipolytica* IMUFRJ 50682, analyzing immobilization yield and microcapsules′ lipase activity.

## 2. Results and Discussion

Gelation is obtained by cross-linking between the carboxyl group of the α-l-guluronic acid of sodium alginate and Ca^2+^ ions. The drop-wise addition of an aqueous solution of sodium alginate and the enzyme in a solution of Ca^2+^ causes the droplets to precipitate and entraps the biocatalyst [13]. Therefore, sodium alginate and CaCl_2_ concentrations used in this process have a huge influence on the entrapment of the enzyme. Chitosan forms polyelectrolyte complexes with alginate, which results in a reinforced gel, reducing leakage and it is usually used as a coating material [13]. In the present work, chitosan was added during gelation to increase its interaction with the matrix, leaving the enzyme to interact with the substrate. However, a high chitosan concentration can reduce mass transfer, decreasing the biocatalyst activity. Therefore, its concentration must also be adjusted. The time used for gelation (complexation time) can also influence the formation of the matrix as an increase in the contact period between these substances may form barriers that reduce diffusional effects. Taqieddin and Amiji [15] reported that increasing the complexation time to more than 5 min did increase the mechanical strength of the beads, but a considerable loss of enzyme activity was observed. As all these variables (sodium alginate, chitosan and calcium chloride concentrations and complexation time) influence each other, a 2^4-1^ fractional factorial design (FFD) was performed to investigate their influence on immobilization yield and on the hydrolytic activity in *p*-nitrophelyl laurate (*p*-NFL) of the microcapsules obtained (immobilized lipase activity—ImLipA). The data obtained are shown in Table 1. The results show that sometimes high immobilization yield (IY) is not accompanied by a high activity of the biocatalyst. This is because it can be effective to retain the enzyme but the pores formed are too small for the substrate to diffuse and, therefore, a low activity is detected.

**Figure 1:**
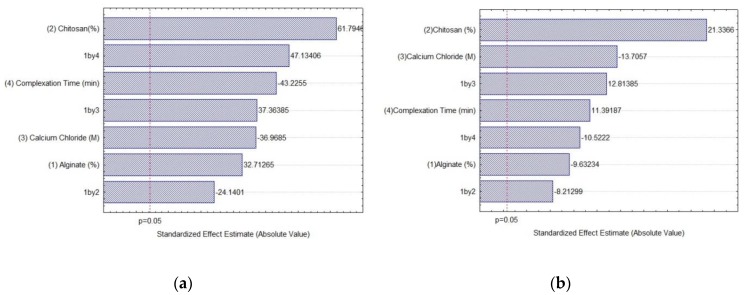
Pareto diagram for the estimated effect of each variable of the 2^4-1^ fractional factorial design: (**a**) Immobilization yield; (**b**) immobilized lipase activity. The point at which the effect estimates were statistically significant (at *p* = 0.05) is indicated by the broken vertical line.

Analysis of variance (ANOVA) and the significance of the results shown in Table 1 were verified using Fisher′s statistical test (*F*-test) at 5% of significance. The results of the analyses can be observed in the Pareto diagrams (Figure 1).

Chitosan was the independent variable with greater influence on immobilization yield and on immobilized lipase activity, with a positive effect (Figure 1), which indicates that higher concentrations of chitosan can improve both responses. High IY were obtained for almost all conditions, but higher ImLipA was only obtained when chitosan was used. On the other hand, negative effects were observed for CaCl_2_ concentration, indicating that its reduction tends to increase the IY and ImLipA. Regarding complexation time, a negative effect was observed for IY and a positive effect for ImLipA, evidencing that reducing the contact time of the reagents favors the immobilization yield, but it does not favor the activity of the biocatalyst. As for alginate concentration, it was found that increasing its concentration may improve IY, but tends to reduce ImLipA. Furthermore, all interactions tested for both responses were also statistically significant.

Since all variables significantly influenced the immobilization yield and the immobilized lipase activity, they were chosen for the optimization strategy using the central composite rotatable design (CCRD). For this design, higher concentrations of chitosan and sodium alginate were used as well as lower calcium chloride concentration and shorter complexation time. Table 2 shows the results obtained and the real values of the levels studied in each experiment.

It is important to observe in Table 2 that IY ranged from 91.76 to 99.77%. Both values were higher than the results from fractional factorial design (FFD) (Table 1), showing the improvement of this response by the statistical designs. The activity of the biocatalyst (ImLipA) ranged from 4.12 to 273.33 U/g, showing also an increase in relation to the FFD, with the highest value (273.33 U/g) almost 14 times higher than for FFD (19.89 U/g). From the experimental values presented, statistical adjustments were made with the purpose of generating significant models. The analysis of variance was performed and the significance of the model verified by Fisher’s statistical test (*F*-test). Tests were performed for the significance of variable effects and for the lack of fit. The complete model was not significant (data not shown) and, therefore, the non-significant terms were eliminated. The results of the variance analysis for the reduced model with IY as response are presented in Table 3.

A significant lack of fit (*p* < 0.05) observed in Table 3 may not be important in the development of a predictive model, according to Rodrigues & Iemma [18], when the pure error, which is associated with the variability of the central points, presents very low value, which is the case here (0.027). Therefore, a mathematical model with the real variables is proposed to represent the immobilization yield (IY) for the studied conditions (Equaion (1)).

IY = 84.852 + 5.017SA − 0.855SA^2^ + 5,865CTS − 26.379CTS^2^ + 61.075CaCl_2_ − 125.082CaCl_2_^2^ − 0.097CT + 0.005CT^2^ + 4.766SA × CTS − 0.028SA × CT − 54.094CTS × CaCl_2_ + 0.478CTS × CT (1)

where SA is sodium alginate concentration, CTS is chitosan concentration, CaCl_2_ is calcium chloride concentration and CT is complexation time.

**Figure 2:**
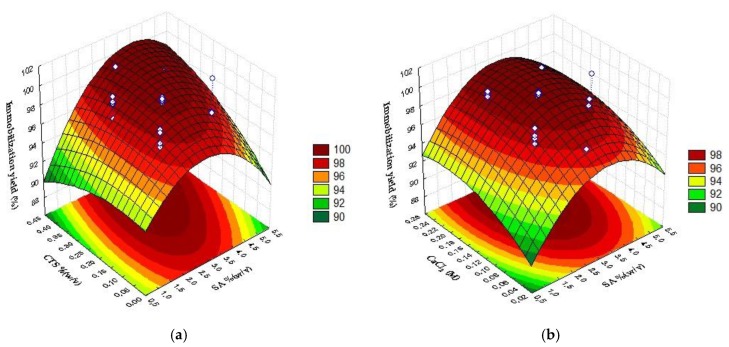

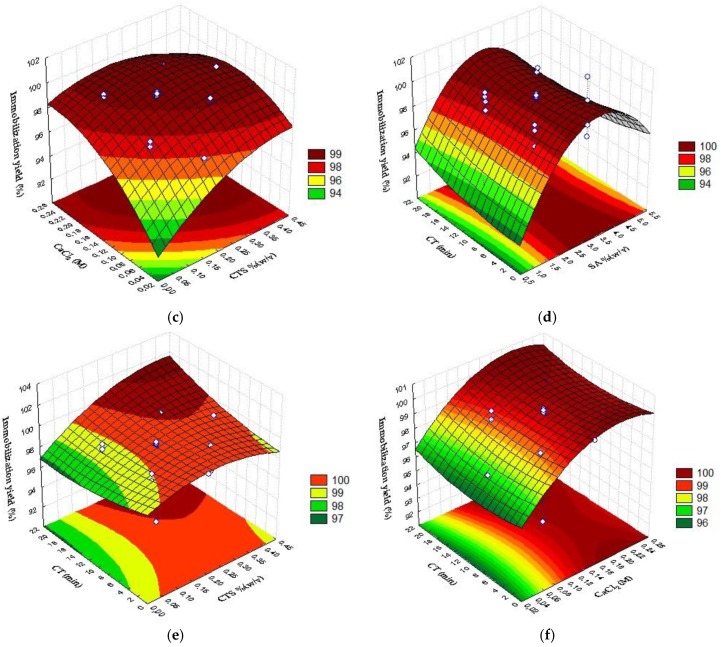
Response surface plots for the immobilization yield, IY (%), as a function of sodium alginate concentration (SA), chitosan concentration (CTS), calcium chloride concentration (CaCl_2_) and complexation time (CT); (**a**) CTS × SA; (**b**) CaCl_2_ × SA; (**c**) CaCl_2_ × CTS; (**d**) CT × SA; (**e**) CT × CTS; (**f**) CT × CaCl_2_.

From the presented model the response surface plots were obtained (Figure 2). The surface plot in Figure 2 shows that in the whole range tested, intermediate values of SA are necessary to increase IY when intermediate to high values of CTS and CaCl_2_ are used (Figure 2a–c). Considering the complexation time, if intermediate values of SA are used (Figure 2d), as well as high values of CaCl_2_ (Figure 2e), high IY are reached independent of the CT chosen. However, high CTS is needed to achieve high values of IY and this condition is obtained with high CT.

Optimum conditions for maximum immobilization yield are: SA, 3.7%(*w*/*v*); CTS, 0.3%(*w*/*v*); CaCl_2_, 0.17 M and CT, 5.4 min. On these conditions, lipase immobilization yield predicted by the model is 100%.

Sodium alginate concentration is determinant for immobilization yield. At low concentrations (<1%) the viscosity of this polymer is low, which can cause internal mixing of the components during complexation, delaying the formation of a semipermeable surface that reduces retention [19]. In the present study higher concentrations (>1%) of this polymer, increased retention (IY), however a small reduction in immobilization yield was observed for SA close to 4%(*w*/*v*) or more, which is associated with viscosity increase. High viscosity makes it difficult to extrude the solution through the syringe needle, preventing uniform formation of microcapsules. Another hypothesis is that when the drops come into contact with the calcium chloride solution, they are influenced by the surface tension difference, forming imperfect microcapsules [20]. These results corroborate studies by Fontes et al. [21] when evaluating the variation of alginate concentration in the microencapsulation of penicillin G in alginate and starch matrix.

Chitosan concentration also exerts a strong influence on the retention of materials by the capsules. Chitosan was used as an alternative to improve immobilization yield by changing the composition of the cationic solution. Chitosan interacts with alginate as a cation reacts with an anion. In our study, chitosan was added to the calcium chloride gelling solution and a remarkable increase in immobilization yield was observed, especially when low alginate concentrations were used. The alginate/chitosan matrix reduced the leaching effect of enzymes owing to the physical and ionic interaction between the enzyme and support, compared to that of using alginate alone. This is because the carboxyl groups of alginate and amino and carboxyl groups of chitosan along with their good hydrophilicity and high porosity create a link with enzymes [22]. According to Peña-Montes et al. [23] the ionic interactions between the enzyme and the support improves the conformational stability of the immobilized enzyme, thereby providing longer shelf life. However, at chitosan concentrations above 0.3% the gelling solution was very viscous for application, causing agglomeration or aggregation of the surface of the microcapsules during formation. Anjani et al. [24] encapsulated flavorzyme and found that when 2.0%(*w*/*v*) alginate and 0.1 M calcium chloride were employed, the immobilization yield was about 18.6%, but when encapsulated in 2.0% alginate and in calcium chloride solution containing chitosan, the immobilization yield increased to 72%, 76% and 84% with 0.1%, 0.2% and 0.3%(*w*/*v*) of chitosan in the cationic solution, respectively.

Calcium chloride is of great importance because it crosslinks with alginate leading to gelation. However, higher Ca^2+^ can reduce the intensity of the ion exchange between chitosan and sodium alginate, reducing the binding capacity of chitosan. It was observed that small concentrations of CaCl_2_ reduce the immobilization yield, which is associated with the small availability of Ca^2+^ ions necessary to the formation of cohesive interactions with alginate, leading to matrix formation. Another variable with great importance on IY is complexation time. CT corresponds to the time required for the diffusion of calcium ions into the microcapsules to promote the crosslinking with the alginate and consequently the formation of the matrix. In our study, it was verified that 5.4 min would be ideal to obtain the maximum efficiency of lipase encapsulation, and it is observed in very long exposure times the leaching of the encapsulated lipase to the calcium chloride solution.

As it was already pointed out, for enzymatic microencapsulation, immobilization yield can be high, but if the capsules do not allow the substrate to diffuse inside, this new biocatalyst presents low activity with no application. Therefore, the analysis of variance was also performed for immobilized lipase activity (ImLipA), as Table 4 shows.

The results presented in Table 4 show that there was no lack of fit (*p* > 0.05), which indicates that the model obtained is adequate for the explanation of the process. In Equation (2) the mathematical model is presented with the real variables, which is proposed to represent the hydrolytic activity of the capsules in *p*-NFL (immobilized lipase activity, ImLipA) under the studied conditions.

ImLipA = 43.92 − 21.50SA + 679.55CTS − 1593.15CTS^2^ + 1470.48CaCl_2_ − 5088.50CaCl_2_^2^ − 14.52CT + 0.59CT^2^ (2)

**Figure 3:**
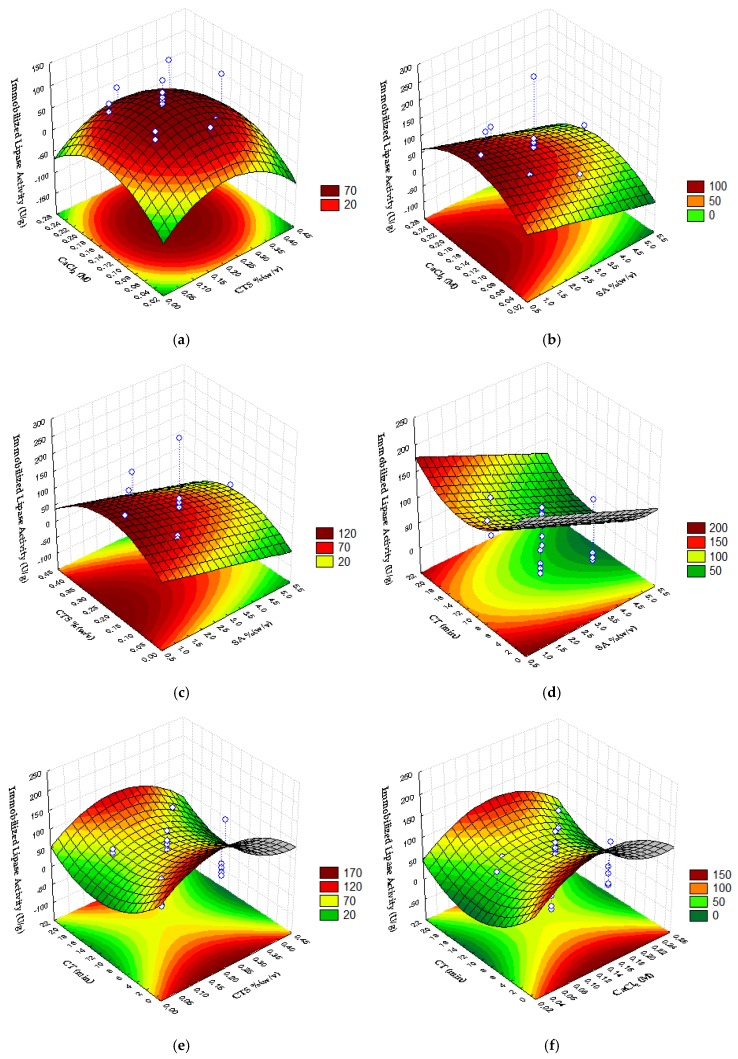
Response surface for the Immobilized Lipase Activity as a function of sodium alginate concentration, chitosan concentration, calcium chloride concentration, and time of complexation; (**a**) CaCl_2_ × CTS; (**b**) CaCl_2_ × SA; (**c**) CTS × SA; (**d**) CT × SA; (**e**) CT × CTS; (**f**) CT × CaCl_2_.

This mathematical model was used to obtain the surface response plots (Figure 3). The hydrolytic activity of the biocatalyst in *p*-NFL is enhanced by intermediate concentrations of CaCl_2_ and CTS (Figure 3a) and at those conditions, SA must be lower (Figure 3b,c). From Figure 3d–f it can be seen that an intermediate complexation time reduces ImLipA, in the range studied.

The increase of sodium alginate concentration may cause conformational changes in the entrapped enzyme and/or can limit substrate mass transfer to the microcapsules [25]. Won et al. [13] studied the immobilization of lipase from *Candida rugosa* and observed a reduction in immobilization yield while increasing alginate concentration. Betigeri and Neau [16] reported that alginate-enzyme interaction reduced lipase activity in relation to beads formed with chitosan. It is possible that the interactions between both polymers (alginate and chitosan) favor enzyme-support interactions that fix the enzyme in the open form, increasing its activity, as for some immobilization strategies reported by Mateo et al. [10].

For chitosan, best results for immobilized lipase activity could be obtained with 0.22%(*w*/*v*). Despite the use of higher CTS to favor immobilization yield, the increase in the amount of this polymer tends to form a diffusional barrier on the surface of the microcapsule, which creates a resistance to the mass transfer of the substrate into the microcapsules, reducing the reaction between enzyme and substrate, directly affecting the immobilized lipase activity.

As for calcium chloride, an optimum value for the hydrolytic activity of the encapsulated enzyme was found when 0.14 M CaCl_2_ was used, which was similar to the one found for the immobilization yield (0.17 M). However, the effect of CaCl_2_ on immobilized lipase activity was small in the range tested (0.05–0.25 M), which may be due to the fact that excess Ca^2+^ to a certain level does not affect matrix formation in the gelation process.

Although better results were observed for CT close to zero, it is worth noting that the contact time of the microcapsules with the cationic solution should be sufficient for the available junction areas to be crosslinked, promoting effective bonds between the alginate and the calcium ions. Roger et al. [26], studying natural magnetic films composed of alginate and maghemite nanoparticles, verified that the ion exchange process is fast, evidencing that half of the conversion takes place in the first 5 min, and after 10 min the concentration of calcium in the film remains constant.

**Figure 4:**
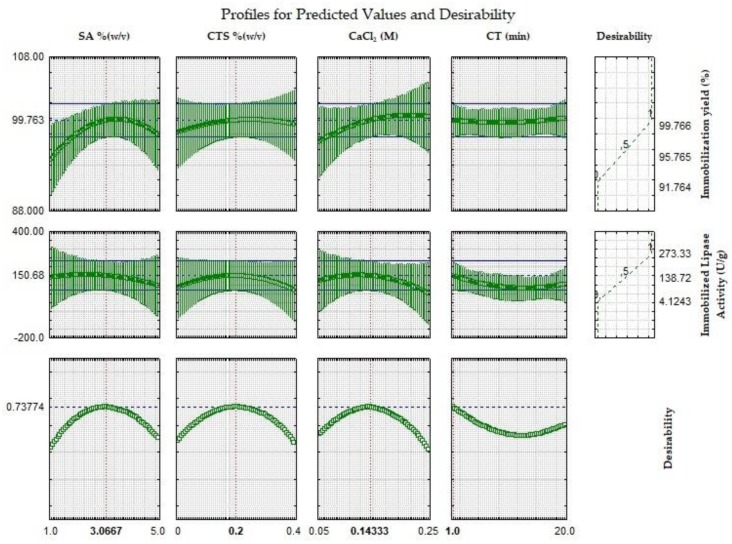
Profile of predicted values and desirability function for immobilization yield and immobilized lipase activity. The dashed vertical line indicates the actual values after optimization.

As both responses (IY and ImLipA) are important for a good biocatalyst, the desirability function was used to optimize the two responses simultaneously. This function is based on a numerical interval that defines the desirability of the analyst in relation to the optimal condition of the process. The range is 0.0–1.0, where (0.0) means unwanted and (1.0) means desired, and the definitions allow to select the most efficient condition for the process in question [27]. Figure 4 shows the results for the optimization of the CCRD through desirability function.

Optimum conditions to obtain 99.8% of immobilization yield and 150.7 U/g of immobilized lipase activity are: sodium alginate concentration of 3.1%(*w*/*v*), chitosan concentration of 0.2%(*w*/*v*), calcium chloride concentration of 0.14 M, and complexation time of 1 min.

**Figure 5:**
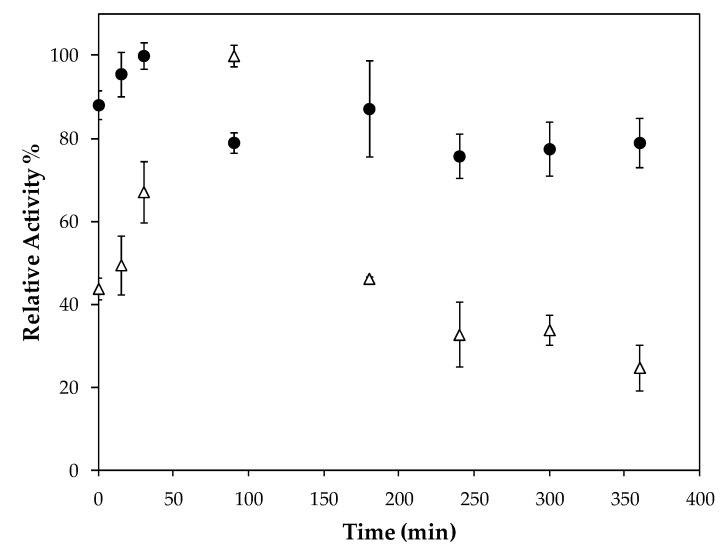
Stability of microcapsules obtained in optimal conditions (3.1%, chitosan concentration of 0.2%, calcium chloride concentration of 0.14 M, and complexation time of 1 min) (●) and free enzyme (LipEE—∆) in hydrolysis reaction with *p*-nitrophenyl laurate at 37 °C, pH 7.0. The enzymatic activity of the highest value for each biocatalyst was set to 100%.

**Figure A1:**
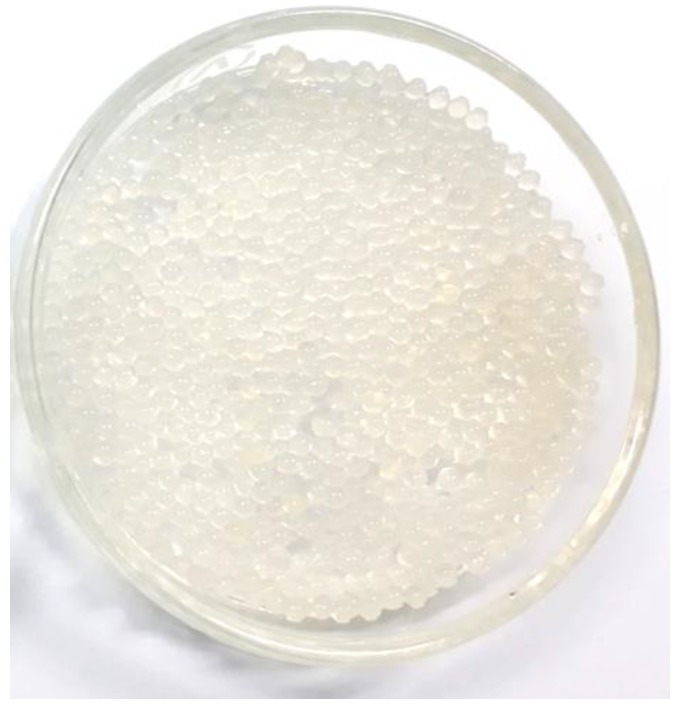
Image of the microcapsules obtained in optimum conditions (sodium alginate concentration of 3.1%(*w*/*v*), chitosan concentration of 0.2%(*w*/*v*), calcium chloride concentration of 0.14 M, and complexation time of 1 min).

A new microencapsulation procedure was performed in those conditions and an IY of 96.7% and ImLipA of 140.0 U/g were obtained for these microcapsules (Appendix A, Figure A1). Considering that 309 U of enzyme activity was encapsulated (by the difference of activity in solution—Appendix A, Table A2) and that 1.78 g (dry weight) of microspheres was produced, an immobilization efficiency of 80.4% was achieved. This biocatalyst was washed and tested for leakage and no significant amount of protein was found in the washing solution or in the leaking solution (Appendix A, Table A2). This biocatalyst presents a higher stability at reaction conditions (37 °C, pH 7.0) than the free enzyme (LipEE), as shown in Figure 5. *C. antartica* lipase B immobilized on green coconut fiber was over 2 times more stable than the free enzyme at 50 °C [28].

This is the first report in literature of of microencapsulation optimization by gelification of lipase from *Y. lipolytica*. The results show the importance of this study since an increase in Immobilization yield to almost 100% and an increase up to 280 U/g of lipase activity of the microcapsules were achieved by using the experimental design optimization tool.

**Figure 6:**
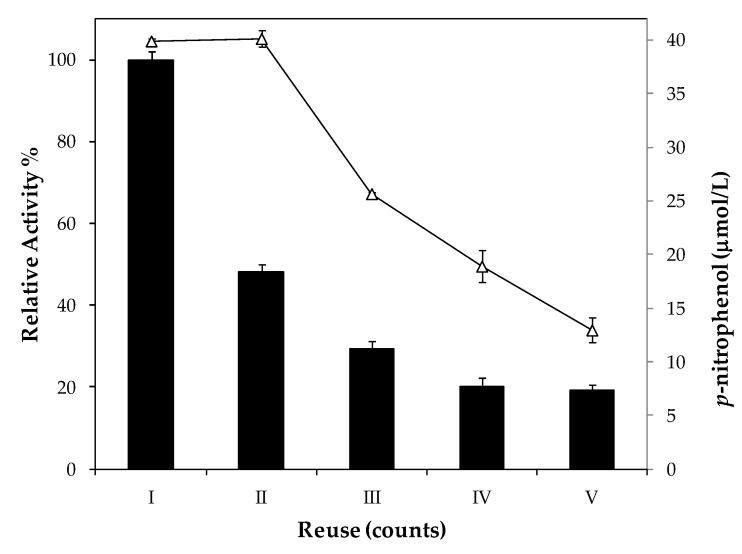
Reusability of microcapsules in hydrolysis reaction with *p*-nitrophenyl laurate as substrate. Bars represent the relative activity of the biocatalyst. The enzymatic activity of the first round was set to 100%. The symbol ∆ represents the amount of product (*p*-nitrophenol) detected after 10 min of hydrolysis.

The reuse of immobilized enzymes is always taken into account for industrial application in order to reduce costs. Thus, the reuse of lipase immobilized by microencapsulation was performed by checking the relative activity of this enzyme over 5 reaction cycles. The microcapsules used for this study were obtained under optimal conditions. Figure 6 shows the observed recycling stability.

The results show that immobilized lipase activity decreased 52% as early as the second reaction cycle and remained with only, approximately, 20% of its initial activity after five reaction cycles. One explanation for this is the leakage of the enzyme from the structure of the microcapsules to the reaction medium. Stolarzewicz et al. [29] reported leakage of cell-bond lipase immobilized in alginate, but in the second cycle only 25% of activity was detected. These microcapsules were tested in a leaking buffer for protein and lipase activity and no protein was detected and just a non-significant activity was determined (Table A2, Appendix A). The measurement of the product (*p*-nitrophenol) considering a 10 min reaction shows that in the second cycle 100% of product is obtained, which reduces with the successive reactions, but not as much as in activity. This indicates that there is a reduction of reaction rate, which might be due to substrate or product retained in the active site. Despite the considerable reduction of the ImLipA, the microcapsules are an interesting alternative for the immobilization of enzymes, since besides protecting them from the external environment, they also have a great advantage in the recovery process. Comparing to other immobilization techniques, alginate microcapsules are easily recycled by simple filtration, dispensing the need to use centrifugation or vacuum filtration.

## 3. Materials and Methods

### 3.1. Materials

Residual frying oil (OFR) used for *Yarrowia lipolytica* lipase production was kindly provided by Brazil Fast Food Corporation. This oil is used to fry potatoes in Bob′s fast food restaurant (Rio de Janeiro, RJ, Brazil). Peptone and yeast extract were purchased from Kasvi (São José dos Pinhais, PR, Brazil) and glucose, calcium chloride, 4-Morpholinepropanesulfonic acid from Vetec (Rio de Janeiro, RJ, Brazil). The sodium alginate, chitosan (medium molecular weight: 190,000–310,000; 200–800 cP, 1 wt. % in 1% acetic acid (25 °C)) and *p*-nitrophenyllaurate (*p*-NFL) were obtained from Sigma Aldrich (St. Louis, MO, USA) and dimethyl sulfoxide was obtained from Isofar (Rio de Janeiro, RJ, Brazil).

### 3.2. Lipase Production

*Yarrowia lipolytica* IMUFRJ 50682, a wild type strain, isolated from Baía de Guanabara, Rio de Janeiro, Brazil [30] was kept at 4 °C on YPD-agar medium. In the inoculum, cells were cultivated at 28 °C in a rotary shaker at 160 rpm, in 500 mL flasks containing 200 mL YPD medium (*w*/*v*: Yeast Extract 1%; Peptone 2%; Glucose 2%) for 72 h. The optical density of a pre-inoculum sample was determined in order to find the initial concentration cells by the dry weight curve. The cells were then centrifuged in 50 mL falcon tubes at 2000× *g* for 5 min and added to the production medium.

Lipase production was carried out in a New Brunswick Microferm MF-114 reactor with an effective volume of 3 L, with 2 mL of antifoam and 3 L of the crude OFR culture medium consisting of residual frying oil, 2.5% (*v*/*v*), peptone, 6.4 g/L and yeast extract 10 g/L [29]. The fermentation was conducted with mechanical agitation (3 Rushton-type stirrers), stirring speed of 650 rpm, aeration rate of 1.5 L·min^−1^, and temperature of 28 °C. Lipase production was performed for 20 h, and the fermented medium was subsequently centrifuged at 4 °C for 10 min at 2000× *g*. Lipase enzymatic extract, herein named LipEE, obtained in cell-free medium was used for the microencapsulation process.

Lipase activity of LipEE was estimated by varying the absorbance at 410 nm in a spectrophotometer (Shimadzu model UV-1800), at 37 °C for 100 s due to the hydrolysis of p-nitrophenyl laurate (p-NFL) [31]. The substrate (p-NFL) was prepared by dissolving 0.018 g of p-NFL in 1 mL dimethyl sulfoxide (DMSO), which was further diluted 100-fold in 50 mM MOPS buffer. One unit is defined as the amount of enzyme that releases 1 mmol of p-nitrophenol per minute at pH 7.0 and 37 °C.

### 3.3. Microencapsulation

**Figure 7:**
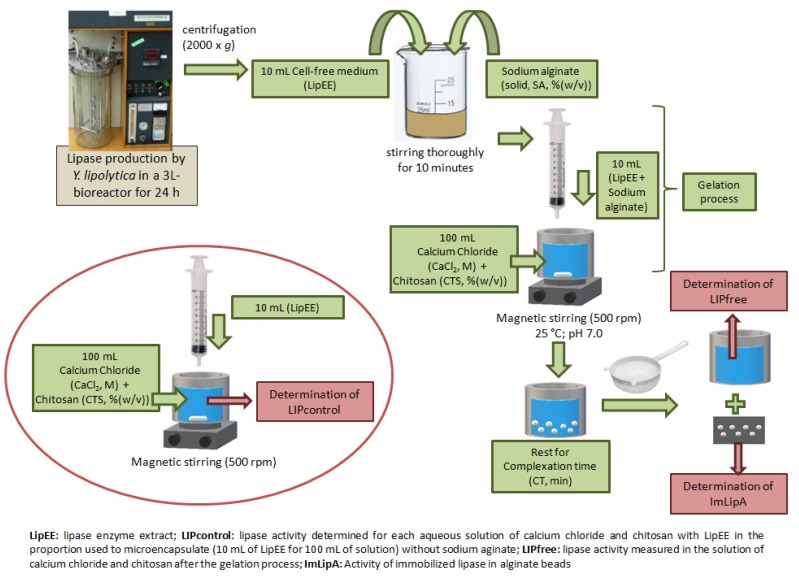
Scheme of microencapsulation process. Sodium alginate concentration (SA), chitosan concentration (CTS), calcium chloride concentration (CaCl_2_) and complexation time (CT).

The formation of microcapsules was carried out using ionotropic pregelation technique [32], with sodium alginate as wall material, as described in Figure 7. Sodium alginate (SA %(*w*/*v*)) was dissolved in LipEE (lipase enzymatic extract) by stirring thoroughly for 10 min to ensure complete mixing. The microcapsules were then formed by dripping 10 mL of biopolymer solution using a 10 mL syringe and 27 g caliber scalp (intravenous infusion device) over 100 mL of an aqueous solution of calcium chloride (CaCl_2_ (M)) and chitosan (CTS %(*w*/*v*)), under stirring (500 rpm) with a magnetic stirrer. Chitosan was first dissolved in a 1%(*w*/*v*) acetic acid solution and sonicated in a 20 kHz horn-type sonicator (Ultrasonic mixing sonicator, DES500, Unique Group, S.P., Brazil) for 8 min. After dissolution, pH was adjusted to 7.0 with NaOH (50%(*w*/*v*)). After gelation, the microcapsules remained in the CaCl_2_ solution for a determined time (complexation time—CT, min). The pH values during the formation of the microcapsules (“complexation time”) were measured and all of them maintained values around 7.0. The microcapsules were then removed with a sieve and lyophilized (Terroni, enterprise 2). Concentrations of sodium alginate (SA, %(*w*/*v*)), calcium chloride (CaCl_2_, M) and chitosan (CTS, %(*w*/*v*)) used in this method, as well as complexation time (CT, min) were optimized in the experimental design.

### 3.4. Optimization of Lipase Microencapsulation

#### 3.4.1. Fractional Factorial Design

A 2^4-1^ fractional factorial design was used to evaluate the significant factors influencing Immobilization yield (IY, %) and Immobilized Lipase Activity (ImLipA, U/g) after drying in a lyophilizer. Four independent variables (Concentrations of sodium alginate (SA, %(*w*/*v*)), calcium chloride (CaCl_2_, M) and chitosan (CTS, %(*w*/*v*)) and complexation time (CT, min)) were included in this study to determine the most significant input factors. Table 1 shows the values representing the levels for each studied parameter. Effects were considered significant for a confidence level of 95% (*p* < 0.05).

#### 3.4.2. Central Composite Design

A central composite rotatable design (CCRD) 2^4^ was defined with the results of the fractional factorial design. Concentrations of sodium alginate (SA, %(*w*/*v*)), calcium chloride (CaCl_2_, M) and chitosan (CTS, %(*w*/*v*)) used in this method, as well as complexation time (CT, min) were the parameters defined and used as independent variables. The limits for each parameter studied are presented in Table 2. Immobilization yield (IY, %) and Immobilized Lipase Activity (ImLipA, U/g) were used as response variables. The results obtained in the experiments were evaluated with Analysis of Variance (ANOVA) and the effects were considered significant when *p* < 0.05. A second-order polynomial model including all linear, quadratic, and linear interaction coefficients was used to calculate the predicted response, as indicated in Equation (3).

(3) $$\mathrm{IY}\;\mathrm{or}\;\mathrm{ImLipA}\;=\;\beta_{0}\;+\;\sum \beta_{i}X_{i}\;+\;\sum \beta_{\mathrm{ii}}X_{i}^{2}\;+\;\sum \beta_{\mathrm{ij}}X_{i}X_{j}\;+\;\sum \beta_{\mathrm{ijk}}X_{i}X_{j}X_{k}$$

where β_0_, β_i_, β_ii_, β_ij_ and β_ijk_ represent the effect of the general process constant, the linear and quadratic effects of X_i_, and the effect of the interaction between X_i_ and X_j_ and X_i_, X_j_ and X_k_ on the encapsulation efficiency and activity of lipase in the microcapsules.

### 3.5. Determination of Lipase Immobilization Yield (IY)

Encapsulation efficiency was determined by quantifying the non-encapsulated lipase activity in the solution of calcium chloride and chitosan after the gelation process. Equation (4) was used to determine the yield.

(4) $$\;\mathrm{IY}\;\left(\%\right)=\frac{\mathrm{LIPcontrol}-\mathrm{LIPfree}}{\mathrm{LIPcontrol}}\;100\;$$

where LIPcontrol is the lipase activity determined for each aqueous solution of calcium chloride and chitosan with LipEE in the proportion used to microencapsulate (10 mL of LipEE for 100 mL of solution) without sodium alginate and LIPfree is the lipase activity measured in the solution of calcium chloride and chitosan after the gelation process. Figure 7 depicts in which stage of microencapsulation process these variables were measured and Table A1 shows the values of LIPcontrol and LIPfree for all conditions tested. Lipase activity was measured as described in Section 3.2.

### 3.6. Determination of Immobilized Lipase Activity

Measurement of immobilized lipase activity was carried out by adding 25 mL of *p*-nitrophenyl laurate (*p*-NFL) solution (prepared as described in Section 3.2) to 10 mg of microcapsules (as shown in Figure 7) obtained as described in Section 3.3. This system was kept under magnetic stirring at 37 °C for 10 min. The reaction was monitored by checking the absorbance (410 nm) in 1-minute intervals for 10 min. One unit is defined as the amount of enzyme that releases 1 mmol of p-nitrophenol per minute at pH 7.0 and 37 °C.

### 3.7. Thermal Stability

Thermal stability was determined by diluting microcapsules with lipase or the cell-free extract (LipEE) in 50 mM sodium phosphate buffer (pH 7) and incubating at 37 °C. The incubation time ranged from 0 to 360 min, after which it was immediately cooled on ice for subsequent residual lipase activity (%) determination as described in 4.6.

### 3.8. Reusability

The reusability was evaluated by measuring the remaining activity of the immobilized lipase after each cycle, and the activity observed in the first cycle defined as 100%. The reactions were performed according to item 4.6, and at the end of each reaction cycle the microcapsules were filtered and a new substrate was added to start a new reaction cycle. Five reuse cycles were performed.

### 3.9. Statistical Analysis

The statistical evaluations were performed with STATISTICA 7.1 software (StatSoft, Inc., Tulsa, OK, USA). The obtained models were statistically verified by means of analysis of variance (ANOVA) and the significance determined by Fisher′s statistical test (*F*-test). The effects were statistically significant when *p* value was less than 0.05. Using surface response methodology, the best conditions for Encapsulation Efficiency and Lipase Activity in Microcapsules were determined for intervals using experimental conditions.

## 4. Conclusions

Maximum immobilization yield for lipase from *Y. lipolytica* was obtained with 3.7% sodium alginate, 0.3% chitosan, 0.17 M calcium chloride and complexation time of 5.4 min. An immobilization yield of 100% was achieved under these conditions, which means a 50% increase in relation to the lowest value accomplished in the range studied. The hydrolytic activity towards *p*-NFL of the microcapsules (immobilized lipase activity—ImLipA) reduced with increasing concentrations of sodium alginate and chitosan was essential for this variable (ImLipA) with 0.22% being the best concentration. Calcium chloride concentration of 0.14 M and complexation times close to zero favored higher values of immobilized lipase activity. To prepare a good biocatalyst a combination of both responses (IY and ImLipA) was considered and for a 99.8% IY and 150.7 U per g of capsules, 3.1% sodium alginate, 0.2% chitosan, 0.14 M calcium chloride and 1-minute complexation time must be used to microencapsulate lipase from *Y. lipolytica*. Microcapsules with lipase from *Y. lipolytica* were more stable at 37 °C than the free enzyme and retained 50% of activity after the second reaction cycle and could be used for five reactions with 20% of initial activity. Therefore, microencapsulation by ionotropic gelation with sodium alginate and chitosan can be considered a good technique for the immobilization of lipase from *Y. lipolytica*.

## Figures and Tables

**Table 1:** Matrix of experimental runs for fractional factorial design (FFD) to evaluate immobilization yield and immobilized lipase activity.

Run	Real Values _(Corresponding Coded Levels)_				IY (%) ^5^	ImLipA (U/g) ^6^
	SA (%) ^1^	CTS (%) ^2^	CaCl_2_ (M) ^3^	CT (min) ^4^		
1	2.00_(−1)_	0.00_(−1)_	0.15_(−1)_	5.0_(−1)_	95.37	1.64
2	4.00_(+1)_	0.00_(−1)_	0.15_(−1)_	25.0_(+1)_	89.83	0.63
3	2.00_(−1)_	0.20_(+1)_	0.15_(−1)_	25.0_(+1)_	94.34	19.89
4	4.00_(+1)_	0.20_(+1)_	0.15_(−1)_	5.0_(−1)_	97.71	4.97
5	2.00_(−1)_	0.00_(−1)_	0.35_(+1)_	25.0_(+1)_	56.92	0.00
6	4.00_(+1)_	0.00_(−1)_	0.35_(+1)_	5.0_(−1)_	89.01	0.00
7	2.00_(−1)_	0.20_(+1)_	0.35_(+1)_	5.0_(−1)_	98.08	2.71
8	4.00_(+1)_	0.20_(+1)_	0.35_(+1)_	25.0_(+1)_	98.71	4.97
9 (C) ^7^	3.00_(0)_	0.10_(0)_	0.25_(0)_	15.0_(0)_	99.19	7.53
10 (C) ^7^	3.00_(0)_	0.10_(0)_	0.25_(0)_	15.0_(0)_	98.63	6.95
11 (C) ^7^	3.00_(0)_	0.10_(0)_	0.25_(0)_	15.0_(0)_	99.21	6.54

^1^ SA: Sodium alginate concentration (%(*w*/*v*)); ^2^ CTS: Chitosan concentration (%(*w*/*v*)); ^3^ CaCl_2_: Calcium chloride concentration (M); ^4^ CT: Complexation time (min); ^5^ IY (%): Immobilization yield; ^6^ ImLipA (U/g): Immobilized Lipase Activity; ^7^ C: central point.

**Table 2:** Matrix of experimental runs for Central composite rotatable design (CCRD) for immobilization yield and immobilized lipase activity.

Run	Real Values _(Corresponding Coded Levels)_				IY (%) ^5^	ImLipA (U/g) ^6^
	SA (%) ^1^	CTS (%) ^2^	CaCl_2_ (M) ^3^	CT (min) ^4^		
1	2.00_(−1)_	0.10_(−1)_	0.10_(−1)_	5.00_(−1)_	97.67	76.92
2	2.00_(−1)_	0.10_(−1)_	0.10_(−1)_	15.00_(+1)_	98.04	58.85
3	2.00_(−1)_	0.10_(−1)_	0.20_(+1)_	5.00_(−1)_	99.43	51.01
4	2.00_(−1)_	0.10_(−1)_	0.20_(+1)_	15.00_(+1)_	99.15	68.74
5	2.00_(−1)_	0.30_(+1)_	0.10_(−1)_	5.00_(−1)_	97.16	41.74
6	2.00_(−1)_	0.30_(+1)_	0.10_(−1)_	15.00_(+1)_	98.76	35.93
7	2.00_(−1)_	0.30_(+1)_	0.20_(+1)_	5.00_(−1)_	98.95	32.66
8	2.00_(−1)_	0.30_(+1)_	0.20_(+1)_	15.00_(+1)_	99.49	110.05
9	4.00_(+1)_	0.10_(−1)_	0.10_(−1)_	5.00_(−1)_	96.51	10.01
10	4.00_(+1)_	0.10_(−1)_	0.10_(−1)_	15.00_(+1)_	95.67	8.15
11	4.00_(+1)_	0.10_(−1)_	0.20_(+1)_	5.00_(−1)_	98.74	4.87
12	4.00_(+1)_	0.10_(−1)_	0.20_(+1)_	15.00_(+1)_	98.67	4.12
13	4.00_(+1)_	0.30_(+1)_	0.10_(−1)_	5.00_(−1)_	98.64	19.12
14	4.00_(+1)_	0.30_(+1)_	0.10_(−1)_	15.00_(+1)_	99.37	12.13
15	4.00_(+1)_	0.30_(+1)_	0.20_(+1)_	5.00_(−1)_	99.56	9.41
16	4.00_(+1)_	0.30_(+1)_	0.20_(+1)_	15.00_(+1)_	99.71	8.22
17	1.00_(−2)_	0.20_(0)_	0.15_(0)_	10.00_(0)_	91.76	124.41
18	5.00_(+2)_	0.20_(0)_	0.15_(0)_	10.00_(0)_	99.36	66.29
19	3.00_(0)_	0.00_(−2)_	0.15_(0)_	10.00_(0)_	96.10	24.99
20	3.00_(0)_	0.40_(+2)_	0.15_(0)_	10.00_(0)_	99.75	82.44
21	3.00_(0)_	0.20_(0)_	0.05_(−2)_	10.00_(0)_	96.88	89.94
22	3.00_(0)_	0.20_(0)_	0.25_(+2)_	10.00_(0)_	98.58	43.18
23	3.00_(0)_	0.20_(0)_	0.15_(0)_	0.00_(−2)_	99.77	273.33
24	3.00_(0)_	0.20_(0)_	0.15_(0)_	20.00_(+2)_	99.14	79.53
25 (C) ^7^	3.00_(0)_	0.20_(0)_	0.15_(0)_	10.00_(0)_	99.41	72.69
26 (C) ^7^	3.00_(0)_	0.20_(0)_	0.15_(0)_	10.00_(0)_	99.50	86.82
27 (C) ^7^	3.00_(0)_	0.20_(0)_	0.15_(0)_	10.00_(0)_	99.64	98.91

^1^ SA: sodium alginate concentration (%(*w*/*v*)); ^2^ CTS: chitosan concentration (%(*w*/*v*)); ^3^ CaCl_2_: calcium chloride concentration (M); ^4^ CT: complexation time (min); ^5^ IY (%): Immobilization yield, IY = (LIPcontrol − LIPfree) × 100/(LIPcontrol); ^6^ ImLipA (U/g): Immobilized Lipase Activity. Table A1 shows the values of LIPfree and LIPcontrol used to calculate IY; ^7^ C: central point.

**Table 3:** Analysis of variance (ANOVA) for central composite rotatable design (CCRD) for immobilization yield.

Factor	DF ^1^	Sum of Square	Mean Square	*F*-Value ^2^	*p*-Value
Sodium Alginate (L)	1	7.483	7.483	559.009	0.00178
Sodium Alginate (Q)	1	15.598	15.598	1165.272	0.00086
Chitosan (L)	1	9.453	9.453	706.200	0.00141
Chitosan (Q)	1	1.485	1.485	110.901	0.00890
Calcium chloride (L)	1	9.727	9.727	726.635	0.00137
Calcium chloride (Q)	1	2.086	2.086	155.839	0.00636
Complexation time (L)	1	0.037	0.037	2.782	0.23727
Complexation time (Q)	1	0.296	0.296	22.076	0.04244
1L by 2L	1	3.635	3.635	271.523	0.00366
1L by 4L	1	0.317	0.317	23.651	0.03978
2L by 3L	1	1.170	1.170	87.439	0.01124
2L by 4L	1	0.913	0.913	68.236	0.01434
Lack of Fit	12	28.458	2.372	177.167	0.00563
Pure Error	2	0.027	0.0134		
Total SS	26	81.210			

^1^ DF: degree of freedom; ^2^ *F*-value: Test for comparing model variance with residual (error) variance. (L) Linear term; (Q) Quadratic term.

**Table 4:** Analysis of variance (ANOVA) for central composite rotatable design (CCRD) for Immobilized Lipase Activity.

Factor	DF ^1^	Sum of Square	Mean Square	*F*-Value ^2^	*p*-Value
Sodium Alginate (L)	1	11098.94	11098.94	64.474	0.0152
Chitosan (L)	1	429.22	429.22	2.493	0.2551
Chitosan (Q)	1	6091.48	6091.48	35.386	0.0271
Calcium chloride (L)	1	188.63	188.63	1.096	0.4051
Calcium chloride (Q)	1	3883.92	3883.92	22.562	0.0416
Complexation time(L)	1	4458.94	4458.94	25.902	0.0365
Complexation time(Q)	1	5219.72	5219.72	30.322	0.0314
Lack of Fit	17	44770.21	2633.54	15.298	0.0630
Pure Error	2	344.29	172.15		
Total SS	26	80519.18			

^1^ DF: degree of freedom; ^2^ *F*-value: Test for comparing model variance with residual (error) variance. (L) Linear term; (Q) Quadratic term.

## Abbreviations

ANOVA	Analysis of Variance
CaCl_2_	Calcium chloride concentration
CCRD	Central Composite Rotatable Design
CT	Complexation Time
CTS	Chitosan concentration
DMSO	Dimethyl Sulfoxide
IY	Immobilization yield
ImLipA	Immobilized Lipase Activity
LipEE	Lipase Enzymatic Extract
OFR	Residual Frying Oil
p-NFL	p-nitrophenyllaurate
SA	Sodium alginate concentration

## Appendix A

**Table A1:** Matrix of experimental runs for Central composite rotatable design (CCRD) for immobilization yield with values of LIPcontrol and LIPfree. IY (%) = (LIPcontrol − LIPfree) × 100/LIPcontrol.

Run	Real Values _(corresponding coded levels)_				LIPcontrol	LIPfree	IY (%) ^5^
	SA (%) ^1^	CTS (%) ^2^	CaCl_2_ (M) ^3^	CT (min) ^4^	(U/L)	(U/L)	
1	2.00_(−1)_	0.10_(−1)_	0.10_(−1)_	5.00_(−1)_	25717.89	599.57	97.67
2	2.00_(−1)_	0.10_(−1)_	0.10_(−1)_	15.00_(+1)_	25717.89	505.13	98.04
3	2.00_(−1)_	0.10_(−1)_	0.20_(+1)_	5.00_(−1)_	31142.95	176.67	99.43
4	2.00_(−1)_	0.10_(−1)_	0.20_(+1)_	15.00_(+1)_	31142.95	263.39	99.15
5	2.00_(−1)_	0.30_(+1)_	0.10_(−1)_	5.00_(−1)_	21001.94	596.28	97.16
6	2.00_(−1)_	0.30_(+1)_	0.10_(−1)_	15.00_(+1)_	21001.94	260.18	98.76
7	2.00_(−1)_	0.30_(+1)_	0.20_(+1)_	5.00_(−1)_	24745.72	259.17	98.95
8	2.00_(−1)_	0.30_(+1)_	0.20_(+1)_	15.00_(+1)_	24745.72	126.81	99.49
9	4.00_(+1)_	0.10_(−1)_	0.10_(−1)_	5.00_(−1)_	16456.84	574.81	96.51
10	4.00_(+1)_	0.10_(−1)_	0.10_(−1)_	15.00_(+1)_	16456.84	711.82	95.67
11	4.00_(+1)_	0.10_(−1)_	0.20_(+1)_	5.00_(−1)_	18558.03	234.61	98.74
12	4.00_(+1)_	0.10_(−1)_	0.20_(+1)_	15.00_(+1)_	18558.03	247.42	98.67
13	4.00_(+1)_	0.30_(+1)_	0.10_(−1)_	5.00_(−1)_	21001.94	285.19	98.64
14	4.00_(+1)_	0.30_(+1)_	0.10_(−1)_	15.00_(+1)_	21001.94	132.39	99.37
15	4.00_(+1)_	0.30_(+1)_	0.20_(+1)_	5.00_(−1)_	24745.72	108.64	99.56
16	4.00_(+1)_	0.30_(+1)_	0.20_(+1)_	15.00_(+1)_	24745.72	72.19	99.71
17	1.00_(−2)_	0.20_(0)_	0.15_(0)_	10.00_(0)_	34048.15	2804.11	91.76
18	5.00_(+2)_	0.20_(0)_	0.15_(0)_	10.00_(0)_	34048.15	218.25	99.36
19	3.00_(0)_	0.00_(−2)_	0.15_(0)_	10.00_(0)_	47419.31	1847.91	96.10
20	3.00_(0)_	0.40_(+2)_	0.15_(0)_	10.00_(0)_	26156.92	65.24	99.75
21	3.00_(0)_	0.20_(0)_	0.05_(−2)_	10.00_(0)_	13701.40	427.32	96.88
22	3.00_(0)_	0.20_(0)_	0.25_(+2)_	10.00_(0)_	13924.64	197.57	98.58
23	3.00_(0)_	0.20_(0)_	0.15_(0)_	0.00_(-2)_	40464.43	94.79	99.77
24	3.00_(0)_	0.20_(0)_	0.15_(0)_	20.00_(+2)_	40464.43	348.09	99.14
25 (C) ^6^	3.00_(0)_	0.20_(0)_	0.15_(0)_	10.00_(0)_	40464.43	239.67	99.41
26 (C) ^6^	3.00_(0)_	0.20_(0)_	0.15_(0)_	10.00_(0)_	40464.43	200.74	99.50
27 (C) ^6^	3.00_(0)_	0.20_(0)_	0.15_(0)_	10.00_(0)_	40464.43	146.46	99.64

^1^ SA: Sodium alginate concentration (%(*w*/*v*)); ^2^ CTS: chitosan concentration %(*w*/*v*); ^3^ CaCl_2_: calcium chloride concentration %(*w*/*v*); ^4^ CT: complexation time (min); ^5^ IY (%): Immobilization yield; ^6^ C: central point.

**Table A2:** Protein and lipase activity determination during the formation of the microcapsules in optimum conditions (sodium alginate concentration of 3.1%, chitosan concentration of 0.2%, calcium chloride concentration of 0.14 M, and complexation time of 1 min).

Solution/Microcapsules	Protein (mg)	Lipase Activity (U)
10 mL LipEE ^1^	1.93 ± 0.07	311.13 ± 33.42
10 mL LipEE + 100 mL (CaCl_2_ solution + chitosan) ^2^	2.12 ± 0.27	319.23 ± 20.89
Aqueous solution after encapsulation (110 mL) ^3^	ND ^6^	10.17 ± 0.52
Microcapsules (1.78 g dry weight)	-	248.52 ± 11.01
100 mL washing solution ^4^	ND ^6^	5.60 ± 0.48
25 mL leaking solution ^5^	ND ^6^	0.48 ± 0.12

^1^ LipEE: Lipase enzyme extract (cell-free medium); ^2^ Control solution: LipEE + calcium chloride and chitosan, without forming the beads (no sodium alginate); ^3^ The aqueous solution obtained after adding 10 mL of LipEE with sodium alginate to 100 mL of calcium chloride solution with chitosan and removing the beads formed after the complexation time; ^4^ distilled water used to wash the beads after formation; ^5^ Buffer solution after immersing the beads for 10 min in phosphate buffer (pH 7.0, 50 mM); ^6^ ND: not detected in the detection range of the method (100–1000 µg/mL).
